# N-myc and STAT interactor is a novel biomarker for predicting the severity and clinical outcome of sepsis: a prospective research

**DOI:** 10.3389/fcimb.2026.1795653

**Published:** 2026-04-27

**Authors:** Xuan Leng, Hui Zhou, Wanying Zhang, Yifei Zeng, Lexin Xia, Chunlu Cheng, Feng Xu

**Affiliations:** 1 Department of Infectious Diseases, The Second Affiliated Hospital, Zhejiang University School of Medicine, Hangzhou, China; 2 Research Center for Life Science and Human Health, Binjiang Institute, Zhejiang University, Hangzhou, China; 3 Key Laboratory of Novel Targets and Drug Study for Neural Repair of Zhejiang Province, School of Medicine, Hangzhou City University, Hangzhou, China

**Keywords:** 30-day mortality, biomarker, disease severity, N-myc and STAT interactor, sepsis, septic shock

## Abstract

**Background:**

Sepsis is prevalent and life-threatening condition that is often associated with high mortality rates. Early stratification is significantly associated with prognosis. Traditional biomarkers show low specificity, while scoring systems are time-consuming. This study aimed to evaluate the predictive value of a novel biomarker (N-myc and STAT interactor (NMI)) in assessing disease severity and clinical outcome in sepsis.

**Methods:**

A total of 399 adult patients diagnosed with sepsis were recruited. The least absolute shrinkage and selection operator (LASSO), logistic regression analysis, and receiver operating characteristic curve (ROC) were used to prove the predictive value of NMI in serum for disease severity (septic shock) and clinical outcome (30-day mortality) in the training set (n = 302). Internal validation (n = 97) was utilized to guarantee the stability of the findings.

**Results:**

The study revealed that NMI was independently associated with 30-day mortality and the occurrence of septic shock through LASSO and logistic analysis. The NMI concentrations of patients with septic shock (166.1 [98.8, 437.5]) and non-survivors (208.7 [113.5, 809.6]) were higher than those of sepsis patients (54.2 [45.4, 64.8]) and survivors (59.3 [48.0, 90.2]), respectively. The area under the curve (AUC) of NMI for predicting mortality and septic shock was 0.86 and 0.92, respectively, which significantly outperformed other biomarkers and scoring systems. The AUCs of new scoring systems containing NMI were all remarkably higher than the original scoring systems (APACHE II, SOFA, qSOFA) for the prediction of clinical outcome and disease severity. Stratification of NMI concentrations in Kaplan-Meier survival curves proved the 30-day survival rate decreased with the increasing level of NMI (*P* < 0.001).

**Conclusions:**

Serum NMI can serve as a novel parameter to predict the severity and clinical outcome of sepsis, potentially facilitating timely disease stratification and providing certain guidance for clinical decision-making.

## 1 Introduction

Sepsis and septic shock are common life-threatening diseases associated with high mortality. According to the recent definition proposed by the Society of Critical Care Medicine (SCCM) and the European Society of Intensive Care Medicine (ESICM), Sepsis-3 has only two grades: sepsis and septic shock, with the concept of severe sepsis removed (Singer et al., 2016). Sepsis is defined as life-threatening organ dysfunction caused by a disorganized immune system to infection. Septic shock is defined as a subtype of sepsis, manifested by circulatory, cellular, and metabolic instability, with a higher mortality risk. Despite significant medical advancements, the mortality rate of sepsis remains alarmingly high. With an estimated 19 to 48.9 million deaths annually, sepsis continues to be a leading cause of death globally and poses a substantial burden on healthcare system (Chiu and Legrand, 2021).

Until now, early evaluation of sepsis remains challenging due to its heterogeneous manifestations and unclear pathogenesis (Timsit and Perner, 2016). Therefore, early identification and stratification are vital so that relevant supportive therapies can be initiated as soon as possible to stop the progression of the disease. The most commonly utilized tools for assessing the severity and mortality of sepsis are scoring systems, including Acute Physiology and Chronic Health Evaluation II (APACHE II) and Sequential Organ Failure Assessment (SOFA) (Tekin et al., 2024). A SOFA score of 2 or more may indicate an overall mortality risk of approximately 10%. Though currently high accuracy in diagnosing and assessing the prognosis of sepsis, the complex procedure of APACHE II is not suitable for dynamic monitoring. The SOFA score, which is simpler than the APACHE II, contains six dimensions and multiple laboratory findings, making it unsuitable for rapid early screening. Quick SOFA (qSOFA) is also widely used for its convenience, as it does not require laboratory tests. However, this convenience comes at the cost of some accuracy in diagnostic and prognostic judgments (Reddy et al., 2024). Therefore, identifying biomarkers that can accurately diagnose and assess the prognosis of sepsis may facilitate precise identification, early intervention, and reduction in mortality (Faix, 2013). However, the predictive values of common biomarkers (C-reactive protein (CRP), procalcitonin (PCT), interleukin-6 (IL-6), *etc*) on mortality of sepsis and the occurrence of septic shock are modest (Suberviola et al., 2012). Accordingly, researchers have been actively seeking novel and specific biomarkers to better evaluate sepsis progression and clinical outcome.

N-myc and STAT interactor (NMI) is an interferon-inducible protein involved in various cellular activities and inflammatory progression (Zhang et al., 2021; He et al., 2022). Zhang et al. used NMI as a new parameter for forecasting the 30-day mortality and intensive care unit (ICU) admission in community-acquired pneumonia (CAP) patients (Zhang et al., 2021). Xiong et al. found NMI was associated with severity and prognosis in acute-on-chronic liver failure of hepatitis B virus (Xiong et al., 2019). Furthermore, the study *in vitro* proved NMI was released by activated macrophages earlier than high-mobility group box protein 1 (HMGB1), which has been considered a typical early biomarker of sepsis (Lu et al., 2014; Xiahou et al., 2017). Weng et al. proved that NMI activated Janus Kinase (JAK) signaling pathway in sepsis, thus upregulating the downstream pro-inflammatory factors and promoting inflammatory responses through analysis of single-cell transcriptome data of sepsis from the Gene Expression Omnibus (GEO) database (Zeng et al., 2024). Knockout of NMI was found to decrease inflammatory responses in murine models of sepsis (Jing et al., 2021). Therefore, we propose that NMI may have predictive value on the early identification of the severity and clinical outcome of sepsis.

However, no studies have yet demonstrated that NMI is highly expressed in the blood of patients with sepsis, and the correlation between NMI and the prognosis of sepsis remains unclear. Therefore, we collected the blood samples from patients suffering from sepsis, and measured the concentrations of NMI to explore whether NMI can serve as a novel molecular marker for predicting the severity and mortality of sepsis.

## 2 Materials and methods

### 2.1 Population

The study was conducted at the Second Affiliated Hospital of Zhejiang University School of Medicine (Hangzhou, Zhejiang, China). This work was approved by the ethics committee. A total of 399 adult patients diagnosed with sepsis, 34 patients with non-septic infection, and 85 healthy controls from the health examination were recruited. All patients and healthy controls were recruited from the Second Affiliated Hospital of Zhejiang University School of Medicine. The 85 healthy controls were recruited from the population undergoing physical examinations and had no discomfort. Patients with non-septic infection were those with infection according to initial vital signs and first medical examination, without showing shock or organ failure features requiring fluid treatment. The 399 adult patients with sepsis were randomly assigned as the training group (n = 302) and the validation group (n = 97) using a computer-generated random number sequence (created with SPSS software) to achieve an approximate 3:1 split, ensuring a completely random and unbiased distribution. All patients met the requirements of the definition of sepsis proposed by ESICM and SCCM (Singer et al., 2016). According to sepsis-3, sepsis is diagnosed based on the presence of confirmed or suspected infection along with an increase in the SOFA score by ≥ 2 points from baseline. The diagnostic criteria for septic shock include hypotension requiring vasopressor support to maintain mean arterial pressure (MAP) > 65 mmHg, plus serum lactate > 2 mmol/L (or >18 mg/dL), when hypovolemia is absent (Srzić et al., 2022). We searched for potential patients through the electronic medical record system of the hospital. Two authors (X.L. and H.Z.) independently made judgments, and in case of inconsistency, the other author (W.Z.) finally decided.

The exclusion criteria for patients and healthy controls were (Singer et al., 2016): (1) age < 18 years old; (2) patients who received chemotherapy, immunosuppressants (including corticosteroids, calcineurin inhibitors, antiproliferative agents, mTOR inhibitors, *etc.*); (3) patients who received hormone therapy within a month before the research; (4) patients with autoimmune diseases; (5) pregnant, or lactating women; (6) patients with hereditary or acquired immunodeficiency; (7) incomplete data (Shin et al., 2021). Additional exclusion criteria for sepsis patients were: (8) patients who had already been diagnosed with sepsis and had received antibiotics at other hospitals; (9) patients with a disease course longer than 24 hours before arriving at our hospital.

### 2.2 Collection of clinical data

The clinical data of sepsis patients, patients with non-septic infection, and healthy controls were obtained from the electronic medical record system of the hospital, including demographics, length of hospital stay, comorbidities, ICU admission, length of stay in ICU, mean arterial pressure (MAP), APACHE II score, SOFA score, qSOFA score, Glasgow coma scale (GCS), life support, sepsis-related complications, operation, sites of primary infection, and clinical outcome. The laboratory test data were also recorded, including arterial blood gas (ABG) analysis, routine blood, CRP, PCT, IL-6, *etc.*

### 2.3 Methods of measurement

All serum samples were collected at the clinical laboratory of the Second Affiliated Hospital of Zhejiang University from March 2024 to March 2025. Peripheral venous blood from the patients was extracted within 48 hours after the diagnosis of sepsis or non-septic infections. Blood samples were stored in a refrigerator of 4°C, and then centrifuged at 1000 rpm for 20 min within 4 hours (Martin et al., 2021). Aliquots of serum were collected and kept at -80°C. The concentrations of NMI were measured by ELISA kits (CSB-EL015893HU, CUSABIO) with instructions. Necessary dilutions ensured that all measured concentrations fell within the sensitivity range of the ELISA kit. The samples below the lower limit were uniformly quantified as half of the minimum detection threshold (11.72 pg/mL). All serum samples were measured in duplicate in a single batch to minimize inter-assay variability. The mean of the measurements was used for analysis. The inter-plate coefficient of variation (CV) was below 10%. Intra-assay CVs were also calculated, and samples with CV > 20% were re-measured.

### 2.4 Statistical analysis

Data with normal distributions were presented as mean ± standard deviation (SD), and data with non-normal distributions were reported as median [25th-75th interquartile range (IQR)]. Categorical data were described as number (percentage). Binary outcome variable data were analyzed by the chi-square test and Fisher’s exact test. For comparing two groups, an independent samples t-test was used when the data were normally distributed, while the Mann-Whitney U test was applied when the data were not normally distributed. For comparing multiple groups, the use of analysis of variance (ANOVA) was appropriate when the data conformed to the assumptions of homoscedasticity and normality, and Tukey’s test or Dunnett’s test was used for multiple pairwise comparisons. Conversely, the Kruskal-Wallis test was used when the data did not conform to these assumptions, and the Nemenyi test was used for multiple pairwise comparisons. The least absolute shrinkage and selection operator (LASSO) and logistic regression were used to identify independent predictors of 30-day mortality and severity of sepsis. The significance of each odds ratio (OR) was assessed using the Wald test. The Kaplan-Meier (KM) analysis was performed to compare the mortality under the stratification of different NMI concentrations. The hazard ratio (HR) was calculated with Cox proportional hazards regression analysis and was tested by the log-rank test. Receiver Operating Characteristic (ROC) curves and areas under the ROC curve (AUCs) were calculated to evaluate the importance of initial NMI concentrations for predicting the severity and clinical outcome of sepsis, and to compare the predictive efficacy of NMI with other biomarkers or scoring systems. The comparison of AUC values was performed using the DeLong test. The integrated discrimination improvement (IDI) and net reclassification improvement (NRI) were also calculated to quantify the added value of NMI to existing scores. *P* < 0.05 was considered statistically significant. All results were analyzed by R Studio (version 4.3.1), GraphPad Prism 8.0 (GraphPad software), and SPSS-16 software.

## 3 Results

### 3.1 Clinical characteristics

The median age of the 302 selected patients in the training group was 66y, with male occupying 67.9% (205/302), and female occupying 32.1% (97/302). The most common coexisting disease was hypertension (49.3%, 149/302), followed by diabetes (28.1%, 85/302) and cancer (23.8%, 72/302). Of the 302 patients, 247 (81.8%) were admitted to the ICU. Within 30 days of sepsis onset, 42.1% (127/302) of the patients died. The detailed data about the training group were described in **Table 1**. The clinical characteristics of survivors and non-survivors, as well as sepsis and septic shock patients, were shown in **Supplementary Tables 1**, **2**.

**Table 1:** Demographics and clinical characteristics of patients in the training group.

Characteristics	Patients with sepsis (n = 302)
Age (y)	66 [57, 75]
Sex	
Male	205 (67.9)
Female	97 (32.1)
LOS (d)	13 [8, 23]
BMI (kg/m^2^)	22.0 [19.1, 24.8]
Complication	265 (87.7)
Diabetes mellitus	85 (28.1)
Hypertension	149 (49.3)
Pulmonary diseases	27 (8.9)
Cardiac diseases	44 (14.6)
Hepatic insufficiency	25 (8.3)
Renal insufficiency	28 (9.3)
Cerebrovascular diseases	52 (17.2)
Cancer	72 (23.8)
MAP (mmHg)	83.2 ± 15.1
GCS	12 [9, 15]
APACHE II	19 [13, 26]
SOFA	9 [6, 11]
qSOFA	1 [0, 1]
Sepsis-related complications	
Septic cardiomyopathy	53 (17.5)
Septic encephalopathy	2 (0.7)
Sepsis-induced MOF	47 (15.6)
Sepsis-induced renal insufficiency	150 (49.7)
Sepsis-induced hepatic insufficiency	99 (32.8)
Sepsis-induced respiratory failure	147 (48.7)
Sepsis-induced coagulation dysfunction	68 (22.5)
Admission into ICU	247 (81.8)
Length of staying in ICU (d)	6 [2, 12]
Operation	113 (37.4)
Life support	216 (71.5)
ECMO	12 (4.0)
Mechanical ventilation	206 (68.2)
CRRT	106 (35.1)
Sites of primary infection	
Blood infection	36 (11.9)
Pulmonary infection	93 (30.8)
Urinary infection	33 (10.9)
Intestinal infection	44 (14.6)
Abdominal infection	59 (19.5)
Biliary infection	9 (3.0)
Other	28 (9.3)
Laboratory indicators	
NMI (pg/mL)	86.6 [55.4, 209.9]
PCT (ng/mL)	7.5 [1.7, 33.8]
CRP (mg/L)	128.0 [66.5, 214.2]
IL-6 (pg/mL)	388.0 [120.8, 1819.0]
Percentage of neutrophils (%)	89.4 [83.1, 92.7]
WBC (× 109/L)	10.7 [6.4, 17.2]
NLR	15.0 [8.4, 25.3]
Lactate (mmol/L)	2.1 [1.3, 3.6]
Septic shock	176 (58.3)
Death	127 (42.1)

Data were shown as n (%), mean ± SD, or median [interquartile range].

APACHE II: acute physiology and chronic health evaluation II. BMI: body mass index. CRP: C-reactive protein. CRRT: continuous renal replacement therapy. ECMO: extracorporeal membrane oxygenation. GCS: Glasgow coma scale. ICU: intensive care unit. IL-6: interleukin-6. LOS: length of stay. MAP: mean arterial pressure. MOF: multiple organ failure. NLR: neutrophil-to-lymphocyte ratio. NMI: N-myc and STAT interactor. PCT: procalcitonin. qSOFA: quick sequential organ failure assessment. SD: standard deviation. SOFA: sequential organ failure assessment. WBC: white blood cell. See also **Supplementary Table 1** and **Supplementary Table 2**.

### 3.2 LASSO and logistic regression of NMI and other variables to predict the severity and 30-day mortality of sepsis

We applied LASSO regression analysis to screen the variables for the best predictors of the model based on a 10-fold cross-validation (Hu et al., 2021). LASSO regression was first used to filter out the independent predictors of septic shock and 30-day mortality. Furthermore, the appropriate tuning parameters (λ) for LASSO regression analysis were confirmed by 10-fold cross-validation. The most important indicators were screened by the LASSO algorithm. Finally, logistic regression analysis was used to analyze the potential relationship between the multiple influencing factors and septic shock or 30-day mortality (Wang et al., 2019).

After performing LASSO regression analysis, univariate analysis and multivariate binary logistic regression analysis were conducted to investigate variables predicting 30-day mortality and septic shock in patients with sepsis. LASSO regression identified SOFA, APACHE II, GCS, septic shock, life support, NMI, PCT, and lactate as potential predictors of 30-day mortality. The univariate analysis corroborated these findings, except for PCT. Further multivariate logistic analysis demonstrated that SOFA, APACHE II, the occurrence of septic shock, life support, NMI, and PCT were independently associated with 30-day mortality. In addition, both LASSO and univariate analysis identified that SOFA, APACHE II, admission into ICU, life support, NMI, and lactate were candidate predictors for septic shock development. However, multivariate logistic analysis revealed that only NMI was independently correlative with the occurrence of septic shock (**Table 2**; **Supplementary Figure 1**).

**Table 2:** LASSO and logistic regression analyses of serum parameters and scores for predicting 30-day mortality and the development of septic shock in the training group.

Variables		Prediction of septic shock				Prediction of mortality			
		Univariate		Multivariate		Univariate		Multivariate	
		OR (95% CI)	*P* value	OR (95% CI)	*P* value	OR (95% CI)	*P* value	OR (95% CI)	*P* value
SOFA		1.314 (1.223-1.421)	**< 0.001**	1.090 (0.984-1.211)	0.104	1.397 (1.290-1.527)	**< 0.001**	1.151 (1.011-1.318)	**0.036**
APACHE II		1.126 (1.089-1.168)	**< 0.001**	1.033 (0.984-1.086)	0.196	1.154 (1.114-1.199)	**< 0.001**	1.064 (1.002-1.131)	**0.044**
GCS		0.845 (0.789-0.900)	**< 0.001**	–	–	0.789 (0.737-0.842)	**< 0.001**	0.966 (0.859-1.083)	0.553
admission into ICU		12.494 (5.931-29.702)	**< 0.001**	2.203 (0.764-6.871)	0.154	24.306 (7.319-150.681)	**< 0.001**	–	–
septic shock		–	–	–	–	23.003 (11.712-49.846)	**< 0.001**	6.396 (2.586-16.936)	**< 0.001**
life support		6.652 (3.847-11.842)	**< 0.001**	1.555 (0.657-3.720)	0.316	23.113 (10.802-91.128)	**< 0.001**	8.663 (2.039-63.066)	**0.010**
NMI (pg/mL)	per 1 pg/mL	1.019 (1.013-1.020)	**< 0.001**	1.014 (1.009-1.020)	**< 0.001**	1.010 (1.007-1.014)	**< 0.001**	1.005 (1.002-1.008)	**0.005**
	per 10 pg/mL	1.208 (1.140-1.223)	**< 0.001**	1.149 (1.088-1.211)	**< 0.001**	1.110 (1.075-1.151)	**< 0.001**	1.046 (1.021-1.085)	**0.005**
PCT (ng/mL)		1.008 (1.001-1.016)	**0.041**	–	–	0.996 (0.989-1.004)	0.330	0.981 (0.968-0.993)	**0.003**
Lactate (mmol/L)		1.312 (1.175-1.494)	**< 0.001**	1.057 (0.957-1.199)	0.317	1.264 (1.157-1.399)	**< 0.001**	1.023 (0.925-1.169)	0.655

APACHE II, acute physiology and chronic health evaluation II; CI, confidence interval; GCS, Glasgow coma scale; ICU, intensive care unit; LASSO, least absolute shrinkage and selection operator; NMI, N-myc and STAT interactor; OR, odds ratio; PCT, procalcitonin; SOFA, sequential organ failure assessment. The bolded text indicated *P* < 0.05. For ease of clinical interpretation, odds ratios were additionally calculated per 10 pg/mL increase in NMI.

### 3.3 The values of NMI predicting the severity and clinical outcome in patients diagnosed of sepsis

**Figure 1:**
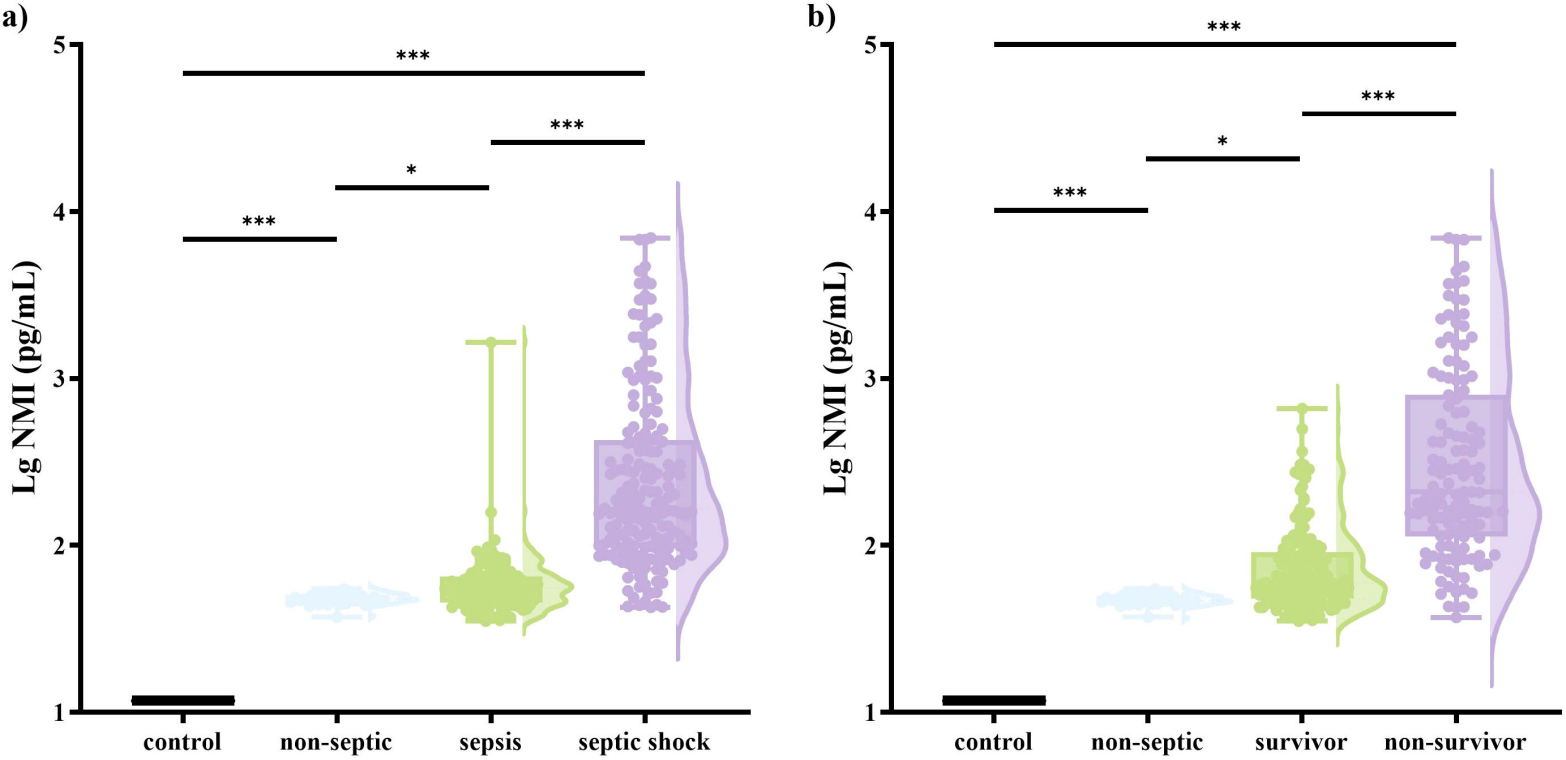
Differences of NMI levels in serum across various groups. **(A)** comparisons among control subjects, patients with non-septic infection, patients with sepsis, and patients with septic shock. **(B)** comparisons among control subjects, patients with non-septic infection, survivors and non-survivors. The samples below the lower limit were uniformly quantified as half of the minimum detection threshold (11.72 pg/mL). NMI, N-myc and STAT interactor. Box plots showed the extremes, medians and quartiles of the data distribution, and violin plots showed the pattern of the data distribution. Statistical comparisons between groups were performed on the raw (untransformed) NMI values.

**Figure 2:**
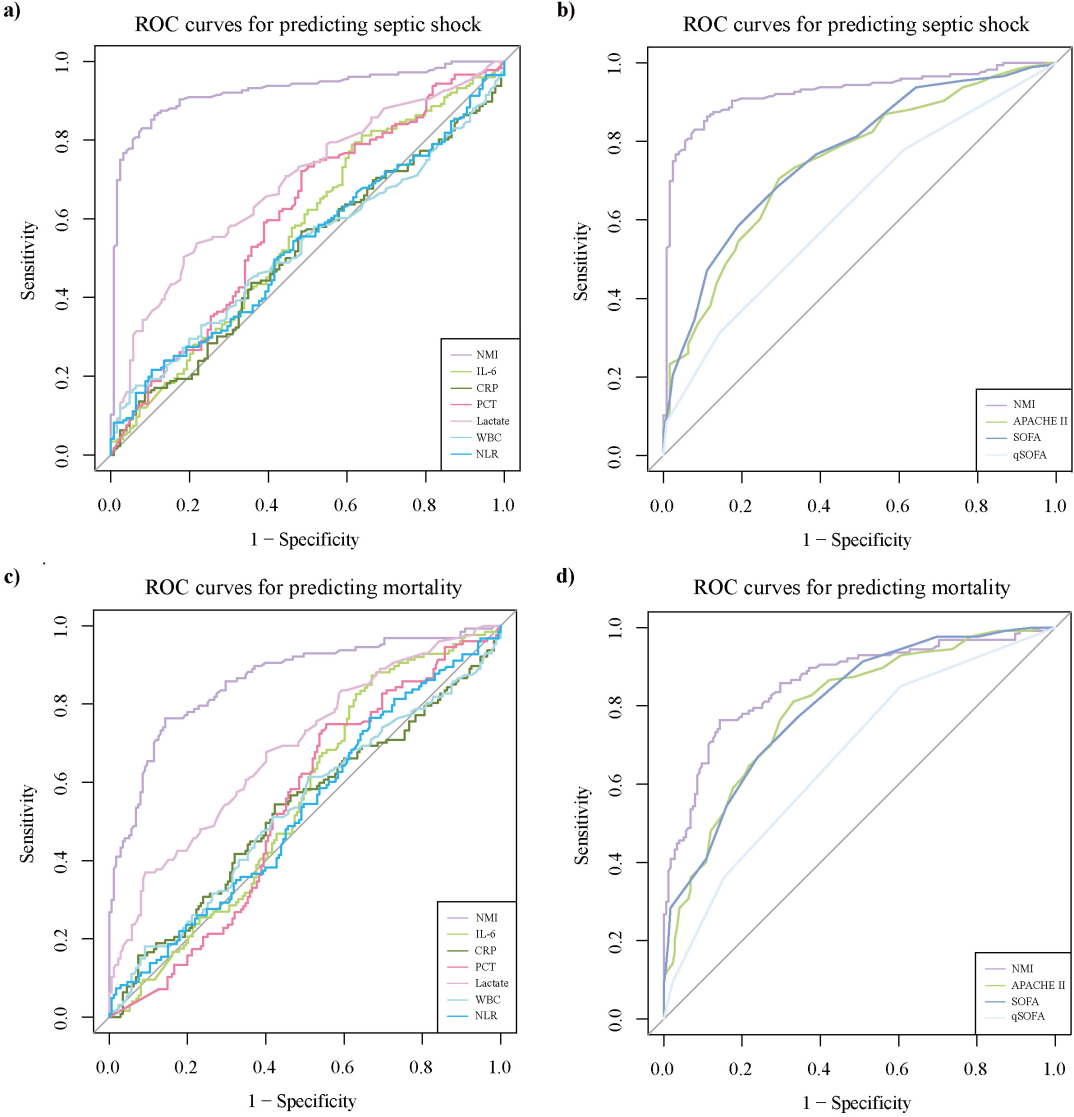
Comparisons between NMI and other serum indicators as well as scores. Comparisons of ROC curves for predicting: **(A)** septic shock development between NMI and other serum parameters; **(B)** septic shock occurrence between NMI and other clinical scores; **(C)** 30-day mortality between NMI and other serum parameters; **(D)** 30-day mortality between NMI and other clinical scores. APACHE II, acute physiology and chronic health evaluation II; CRP, C-reactive protein; IL-6, interleukin-6; NLR, neutrophil-to-lymphocyte ratio; NMI, N-myc and STAT interactor; PCT, procalcitonin; qSOFA, quick sequential organ failure assessment; ROC, receiver operating characteristic; SOFA, sequential organ failure assessment; WBC, white blood cell.

Patients with septic shock exhibited significantly higher scores of qSOFA, SOFA, and APACHE II, as well as higher levels of NMI, lactate, and PCT compared to those with sepsis (**Supplementary Table 1**). Except for PCT, these indicators were also elevated in non-survivors (**Supplementary Table 2**). Additionally, other common inflammatory markers, such as CRP and IL-6, showed numerically higher levels in patients with septic shock and non-survivors, though these differences did not reach statistical significance (**Supplementary Tables 1**, **2**). Compared with healthy controls (11.7 pg/mL) and patients with non-septic infection (47.1 pg/mL), the level of NMI (86.6 pg/mL) was markedly increased (**Supplementary Table 3**; **Figure 1**). The NMI concentrations of patients with septic shock (166.1 [98.8, 437.5]) and non-survivors (208.7 [113.5, 809.6]) were significantly higher than those of patients with sepsis (54.2 [45.4, 64.8]) and survivors (59.3 [48.0, 90.2]), respectively (**Supplementary Tables 1**, **2**; **Figure 1**). In the training group, the AUC of NMI for predicting the occurrence of septic shock was 0.92 (95%CI: 0.89-0.96), significantly higher than that of other parameters and scores (**Table 3**; **Figures 2A, B**). The corresponding cut-off value was 80.45 pg/mL. Similarly, the AUC of NMI for predicting 30-day mortality was 0.86 (95%CI: 0.82-0.90) (**Table 3**; **Figures 2C, D**), outperforming other indicators. The cut-off value for this prediction was 110.85 pg/mL.

**Table 3:** Diagnostic performance analyses of serum indicators and scores for sepsis patients in the training group.

Variables	Prediction of septic shock					Prediction of 30-day mortality				
	Cut-off value	AUC (95% CI)	Specificity (%)	Sensitivity (%)	Youden index	Cut-off value	AUC (95% CI)	Specificity (%)	Sensitivity (%)	Youden index
NMI (pg/mL)	80.45	0.92 (0.89-0.96)	88.9	86.4	0.75	110.85	0.86 (0.82-0.90)	85.7	76.4	0.62
IL-6 (pg/mL)	151.80	0.56*** (0.50-0.63)	38.1	79.0	0.17	120.50	0.55*** (0.49-0.62)	32.6	87.4	0.20
CRP (mg/L)	120.25	0.51*** (0.45-0.58)	51.6	56.8	0.08	132.40	0.53*** (0.46-0.60)	57.7	54.3	0.12
PCT (ng/mL)	4.26	0.61*** (0.50-0.67)	51.6	72.2	0.24	3.98	0.54*** (0.47-0.60)	44.6	74.8	0.19
Lactate (mmol/L)	2.41	0.69*** (0.63-0.75)	77.0	54.0	0.31	4.75	0.68*** (0.62-0.74)	90.9	37.0	0.28
WBC (× 10^9^/L)	22.55	0.53*** (0.46-0.59)	93.7	17.6	0.11	9.95	0.53*** (0.46-0.60)	49.1	61.4	0.11
NLR	32.86	0.53*** (0.47-0.60)	89.7	21.0	0.11	9.57	0.53*** (0.46-0.60)	33.1	77.2	0.10
APACHE II	18.50	0.75*** (0.69-0.80)	70.6	70.5	0.41	18.50	0.79* (0.74-0.84)	66.9	81.1	0.48
SOFA	9.50	0.77*** (0.71-0.82)	81.0	58.5	0.40	9.50	0.80* (0.75-0.85)	76.0	66.9	0.43
qSOFA	1.50	0.67*** (0.61-0.73)	85.7	31.2	0.17	0.50	0.70*** (0.61-0.73)	39.4	85.0	0.24

APACHE II, acute physiology and chronic health evaluation II; AUC, area under the curve; CI, confidence interval; CRP, C-reactive protein; IL-6, interleukin-6; NLR, neutrophil-to-lymphocyte ratio; NMI, N-myc and STAT interactor; PCT, procalcitonin; qSOFA, quick sequential organ failure assessment; SOFA, sequential organ failure assessment; WBC, white blood cell. *P < 0.05, **P < 0.01, ***P < 0.001 compared with NMI.

**Figure 3:**
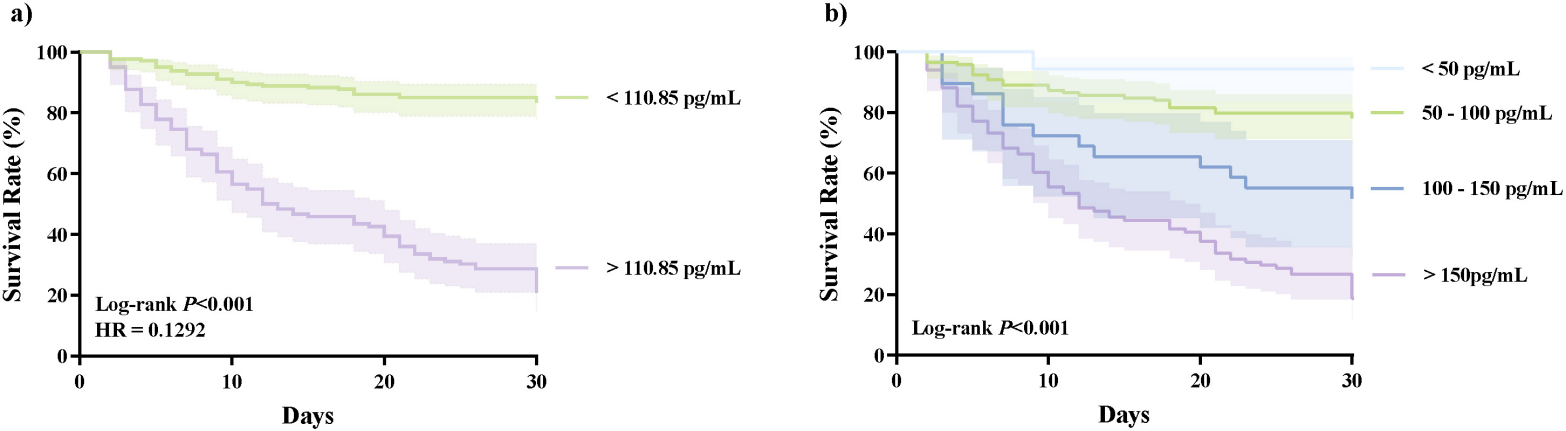
Kaplan-Meier survival curves created by NMI cut-off value and concentration stratification for forecasting the survival rate. **(A)** the Kaplan-Meier survival curve created by the cut-off value (110.85 pg/mL). **(B)** the Kaplan-Meier survival curve created by the stratification of NMI concentrations (< 50 pg/mL, 50–100 pg/mL, 100–150 pg/mL, > 150 pg/mL). HR, hazard ratio; NMI, N-myc and STAT interactor.

Furthermore, we drew KM survival curves to describe the difference in 30-day mortality stratified by the NMI concentrations (**Figure 3**). Patients with NMI levels above the cut-off value (110.85 pg/mL) exhibited a markedly lower 30-day survival rate (*P* < 0.001) (**Figure 3A**). Further stratification of NMI concentrations also proved the 30-day survival rate decreased with the increasing level of NMI (**Figure 3B**).

### 3.4 The added values of NMI for existing scores to predict the severity and mortality of sepsis patients

**Figure 4:**
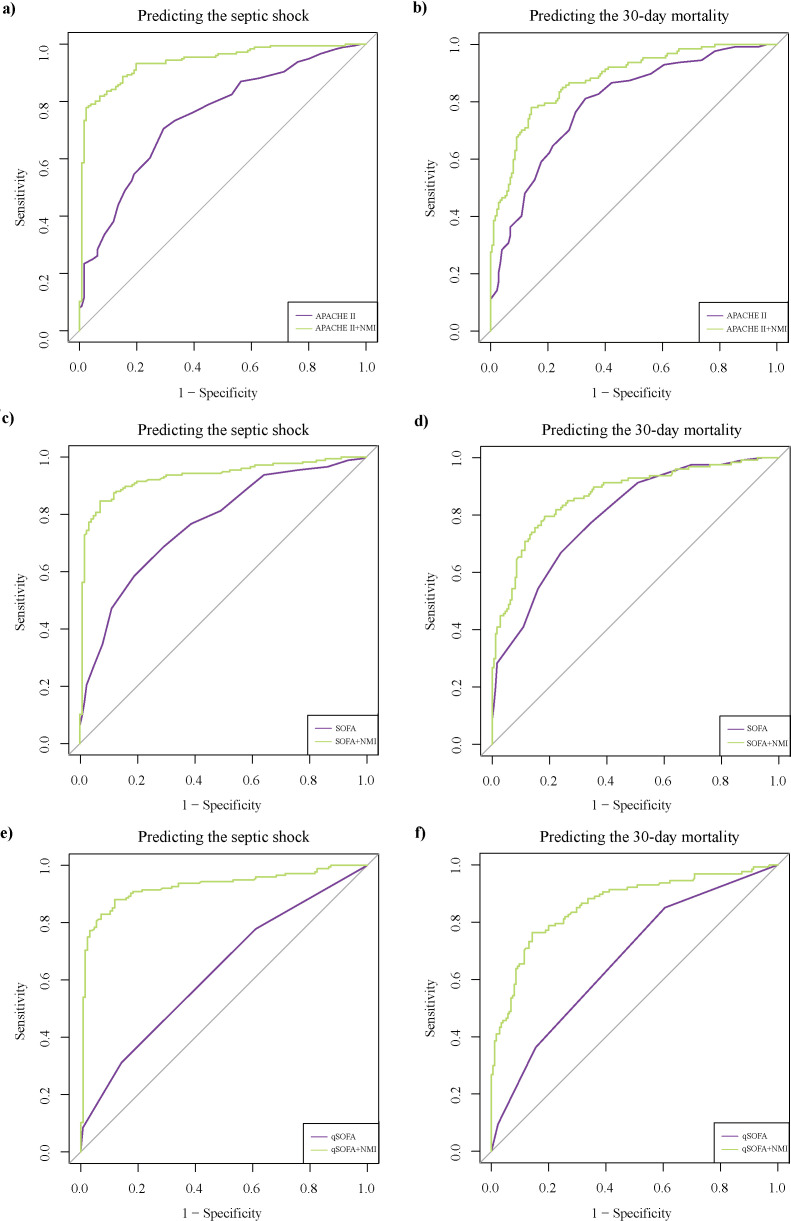
Additional values of NMI on scores for predicting septic shock and 30-day mortality. Comparison of ROC curves between APACHE II and the new score system containing NMI for predicting the severity **(A)** and outcome **(B)** of sepsis. ROC curves between SOFA and the new score system containing NMI for predicting the severity **(C)** and outcome **(D)** of sepsis. ROC curves between qSOFA and the new score system containing NMI for predicting the severity **(E)** and outcome **(F)** of sepsis. APACHE II, acute physiology and chronic health evaluation II; NMI, N-myc and STAT interactor; qSOFA, quick sequential organ failure assessment; ROC, receiver operating characteristic; SOFA, sequential organ failure assessment.

We further evaluated whether the addition of NMI would help improve the predictive efficiency of existing rating systems (APACHE II, SOFA, qSOFA) for disease severity and clinical outcome in the training cohort. Two methods were employed. First, we calculated the NRI and IDI to verify the improving effect of NMI on scores. NRI is used to compare the difference in classification accuracy between two prediction models, while IDI is often applied to evaluate the overall improvement of the prediction probability of the new model compared to the old model. The IDI or NRI > 0 represents that the new model has a positive improvement compared to the old model (Vickers et al., 2016). Both NRI and IDI showed that the performance of scores for predicting the development of septic shock and 30-day mortality could be significantly enhanced by NMI (**Table 4**). On the other hand, we compared the AUCs of ROC curves between primary rating systems and new systems containing NMI. For APACHE II, the new rating system (NMI+APACHE II) exhibited superior performance in predicting both the development of septic shock (AUC 0.94 vs. 0.75, *P* < 0.001) and the 30-day mortality (AUC 0.88 vs. 0.79, *P* < 0.01) (**Figures 4A, B**; **Supplementary Table 4**). Similarly, the AUC of the NMI + SOFA system was significantly higher than that of the single SOFA system for predicting both disease severity (AUC 0.93 vs. 0.77, *P* < 0.001) and mortality (AUC 0.87 vs. 0.80, *P* < 0.05) (**Figures 4C, D**; **Supplementary Table 4**). The same trend was observed with the NMI+qSOFA compared to the qSOFA system alone. The AUC for predicting septic shock was 0.93 vs. 0.67 (*P* < 0.001), and for predicting mortality, it was 0.86 vs. 0.70 (*P* < 0.001) (**Figures 4E, F**; **Supplementary Table 4**). These results highlight the enhanced predictive power of 30-day mortality and disease severity, when integrating NMI into existing scoring systems.

**Table 4:** The supplementary values of NMI to other scores for predicting the severity and outcome of sepsis in the training group.

Indicators	Predicting the septic shock			Predicting the 30-day mortality		
	APACHE II	SOFA	qSOFA	APACHE II	SOFA	qSOFA
NRI (95% CI)	0.63*** (0.50-0.75)	0.57*** (0.45-0.70)	0.73*** (0.59-0.86)	0.40*** (0.26-0.53)	0.39*** (0.28-0.51)	0.75*** (0.61-0.88)
IDI (95% CI)	0.27*** (0.23-0.30)	0.24*** (0.21-0.28)	0.35*** (0.31-0.38)	0.21*** (0.16-0.26)	0.19*** (0.15-0.23)	0.28*** (0.24-0.32)

APACHE II, acute physiology and chronic health evaluation II; CI, confidence interval; IDI, integrated discrimination improvement; NMI, N-myc and STAT interactor; NRI, net reclassification improvement; qSOFA, quick sequential organ failure assessment; SOFA, sequential organ failure assessment. ***P < 0.001.

### 3.5 Results of internal verification

The baseline data between the validation group and the training group were observed no significant difference (**Supplementary Table 5**). In the validation group, the AUC of ROC for predicting septic shock was 0.86 (95%CI: 0.79-0.94, specificity 92.3%, sensitivity 69.0%), with the cut-off at 86.89 pg/mL (**Supplementary Figure 2A**). The AUC for predicting 30-day mortality was 0.92 (95%CI: 0.87-0.97, specificity 89.1%, sensitivity 83.3%), with the cut-off at 87.12 pg/mL (**Supplementary Figure 2B**). The above results were similar to those of the training group, which proved the stability and accuracy of the predictive efficiency of NMI.

## 4 Discussion

Despite the advancement of therapeutic methods, the mortality of sepsis patients still remains high, with the subtype septic shock reaching 30% to 50% (Coopersmith et al., 2012). Thus, the management of sepsis and septic shock should be taken as an emergency. Screening patients with sepsis can facilitate early medical intervention (Cecconi et al., 2018; Evans et al., 2021). High-quality biomarkers can be rapidly measured and used as indicators to accurately predict the development and clinical outcome of sepsis. This capability facilitates rapid risk stratification and provides valuable guidance for clinical decision-making. Our study identified NMI as a promising predictor of sepsis progression and mortality.

Some biomarkers, such as CRP, PCT, and IL-6, have been proposed to assist the evaluation of sepsis. However, the role of these indicators in the risk stratification of septic shock remains unclear. In addition, there are few credible biomarkers that can perform well in predicting the prognosis and clinical outcome of sepsis (Kong et al., 2023; He et al., 2024). A study by Aliu-Bejta A et al. revealed that there was no statistically significant difference in the concentrations of CRP and PCT between the sepsis and septic shock patients (Aliu-Bejta et al., 2020). The same condition was observed at the concentrations of CRP and WBC, in the research of Yamamoto T et al (Yamamoto et al., 2019). The severity-predictive value of IL-6 has also remained inconsistent and controversial. Some studies proved higher IL-6 concentrations in patients diagnosed with septic shock compared to sepsis patients, while other studies have reported completely opposite results (Tschaikowsky et al., 2011; Mat-Nor et al., 2016; Hamed et al., 2017; Song et al., 2019). Consistent with previous research, the CRP, PCT, IL-6, NLR, and WBC in our study showed no significant difference between the non-survivors and survivors. None of the above parameters except PCT showed differences between patients with sepsis and septic shock (**Supplementary Tables 1**, **2**).

On the other hand, common clinical scores used to evaluate sepsis, such as SOFA and APACHE II, seem to have limitations in application, mainly due to their time-consuming trait. The use of qSOFA has been recommended for a rapid evaluation of sepsis, with a little sacrifice of specificity and accuracy (Singer et al., 2016). Therefore, several biomarkers have been proposed to improve the accuracy of existing score systems, accelerate the assessment process, and help stratify septic shock patients (Castello et al., 2019; Chen et al., 2020; Wu et al., 2025). Both the index of NRI as well as IDI, and the comparison of AUC of ROC curves, proved that NMI had supplementary values for common score systems (APACHE II, SOFA, qSOFA) in predicting the severity and clinical outcome in sepsis (**Table 4**; **Figure 4**).

The infiltration of inflammatory cells and the activation of immune-related pathways are typical features of sepsis and septic shock (Bosmann and Ward, 2013; Ye et al., 2021). It is widely acknowledged that damage-associated molecular patterns (DAMPs), including NMI, can upregulate the release of inflammatory indicators through the activation of Toll and NF-κB pathway in sepsis (Zeng et al., 2024). Ouyang et al. found that NMI could exacerbate the infection of influenza A by increasing degradation of interferon regulatory factor 7 (IRF7) via tripartite motif 21 (Ouyang et al., 2021). Researches have demonstrated that NMI can activate nuclear factor kappa-B (NF-κB) in macrophages through Toll-like receptor (TLR) 4 pathway, thereby inducing the release of proinflammatory factors such as IL-6 (Qiang et al., 2013; Xiahou et al., 2017; Ouyang et al., 2021). Soluble extracellular NMI can directly act on peripheral monocytes and stimulate the release of inflammatory cytokines by targeting plasmalemmal TLR4 (De Masi et al., 2021). Correspondingly, NMI knockout (NMI^-/-^) alleviated inflammatory response and improved the survival rate in lipopolysaccharide (LPS)-induced sepsis models. Transcriptome data analysis also confirmed significantly upregulated NMI expression in sepsis (Zeng et al., 2024). These studies demonstrate that the involvement of NMI in the progression of inflammation in sepsis. Moreover, a study found that the NMI was released by macrophages rather than pyroptotic cells, indicating that the discharge of NMI occurred much earlier than tissue damage (Xiahou et al., 2017; Liang and He, 2022). The study observed that NMI was released earlier than HMGB1, with the latter a classical marker of the early stage in sepsis. Besides, the fact that NMI levels were directly associated with the mortality of patients who succumbed to severe inflammation was also observed. All studies enhance the credibility that NMI may serve as an early molecular parameter for the evaluation of sepsis. In this study, the concentrations of NMI in non-survivors and patients with septic shock were both higher than those in survivors and sepsis patients, respectively (**Supplementary Tables 1**, **2**; **Figure 1**). LASSO and multivariate regression also demonstrated that NMI was an independent parameter of both the development of septic shock and 30-day mortality (**Table 2**). Higher levels of NMI were associated with a lower survival rate (**Figure 3**). The above results demonstrated that NMI could serve as a novel biomarker for predicting 30-day mortality and disease severity. Compared to previous biomarkers such as monocyte-derived matrix metalloproteinase-9 (MMP9) and Enah/Vasp-like (EVL), NMI offered significant advantages for clinical translation. Based on clinical samples, this study not only confirmed the independent predictive value of NMI for septic shock and mortality, but also demonstrated that integrating NMI into existing clinical scoring systems significantly improved their predictive performance. This provided a simple and feasible new strategy for optimizing clinical risk stratification (Ning et al., 2023a; Ning et al., 2023b; Wang et al., 2025b; Wang et al., 2025a). In clinical practice, early high concentrations of NMI represented a higher mortality rate and a higher probability of developing septic shock, indicating that clinical doctors need to be alert to the rapid deterioration of the disease and take corresponding measures as soon as possible.

It is worth noting that the lactate levels also showed significant differences between the two groups, respectively. Serum concentrations of lactate are classically deemed as an indicator of tissue hypoxia and applied as a biomarker of the severity and clinical outcome of patients diagnosed with sepsis (Nolt et al., 2018). Previous studies unveiled that high circulating levels of lactate were correlated with the high severity and mortality of sepsis. Lactate can also promote the lactylation and exosomal release of HMGB1 (Yang et al., 2022). Considering that HMGB1 is a currently recognized parameter for early identification of sepsis, lactate is also widely studied to assess its prognostic and severity predictive accuracy (Weinberger et al., 2021). The results in our study were consistent with past studies, but the predictive performance of lactate was inferior to that of NMI, as proven by ROC curves. On the other hand, we also attempted to explore the ability of NMI to distinguish between sepsis and non-septic infection. It seemed NMI also displayed excellent performance in discriminating sepsis from non-septic infection. However, given the absence of a validation cohort and insufficient cases, these results should be interpreted with caution. Further investigation with larger and more balanced samples is warranted.

The study also had several limitations. First, the high specificity of the biomarker should be interpreted with caution because the research was conducted in a sepsis-specific setting. Second, the study sample size was relatively small and participants were recruited from a single institution, which may limit the generalizability of the findings. Finally, although NMI demonstrated favorable diagnostic performance, the biological kinetics of circulating NMI remain incompletely characterized. As an interferon-inducible intracellular protein, its presence in serum likely reflects inflammatory activation or cellular injury; however, data regarding its stability and potential influence of pre-analytical handling are limited. All samples in this study were processed under a standardized protocol to minimize variability. Future studies are warranted to further evaluate the stability and dynamic changes of circulating NMI in clinical settings.

## 5 Conclusion

In this study, serum NMI levels were found to be remarkably elevated in severe patients with sepsis at an early stage. The study demonstrated that NMI may serve as a novel biomarker for predicting 30-day mortality and disease severity, thereby assisting early severity stratification and timely clinical decision-making for patients with sepsis.

## Nomenclature

ABG, arterial blood gas; ANOVA, analysis of variance; APACHE II, Acute Physiology and Chronic Health Evaluation II; AUC, areas under the ROC curve; BMI, body mass index; CAP, community-acquired pneumonia; CI, confidence interval; CRP, C-reactive protein; CRRT, continuous renal replacement therapy; CV, coefficient of variation; DAMP, damage-associated molecular pattern; ECMO, extracorporeal membrane oxygenation; ESICM, European Society of Intensive Care Medicine; EVL, Enah/Vasp-like; GCS, Glasgow coma scale; GEO, Gene Expression Omnibus; HMGB1, high-mobility group box protein 1; HR, hazard ratio; ICU, intensive care unit; IDI, integrated discrimination improvement; IL-6, interleukin-6; IRF7, interferon regulatory factor 7; JAK, Janus Kinase; KM, Kaplan-Meier; LASSO, least absolute shrinkage and selection operator; LOS, length of stay; LPS, lipopolysaccharide; MAP, mean arterial pressure; MMP9, monocyte-derived matrix metalloproteinase-9; MOF, multiple organ failure; mTOR, mechanistic target of rapamycin; NF-κB, nuclear factor kappa-B; NLR, neutrophil-to-lymphocyte ratio; NMI, N-myc and STAT interactor; NRI, net reclassification improvement; OR, odds ratio; PCT, procalcitonin; qSOFA, quick SOFA; ROC, receiver operating characteristic curve; SCCM, Society of Critical Care Medicine; SD, standard deviation; SOFA, Sequential Organ Failure Assessment; TLR, Toll-like receptor; WBC, white blood cell.

## Data Availability

The datasets presented in this article are not readily available because all data generated and analyzed during the study have been included in the manuscript. The original datasets are not publicly available due to institutional ethics, privacy, and confidentially regulations, but are available from the corresponding author on reasonable request. Requests to access the datasets should be directed to xufeng99@zju.edu.cn.
