# Supplementary material for: N-myc and STAT interactor is a novel biomarker for predicting the severity and clinical outcome of sepsis: a prospective research
Source: Front Cell Infect Microbiol. 2026 Apr 27;16:1795653. doi: 10.3389/fcimb.2026.1795653 (PMC13158202; doi:10.3389/fcimb.2026.1795653)
Supplement: Supplementary file 1 [file Table1.docx]

Supplementary Material

# Supplementary Results

## The values of NMI distinguishing between sepsis and non-septic infection

We also attempted to explore the ability of NMI to distinguish between sepsis and non-septic infection. The AUC reached 0.85 (95% CI: 0.81-0.89, specificity 76%, sensitivity 100%). The corresponding cut-off value was 55.17 pg/mL. The result showed that NMI also displayed excellent performance in discriminating sepsis from non-septic infection (Supplementary Figure 3).

# Supplementary Figures and Tables

## Supplementary Figures


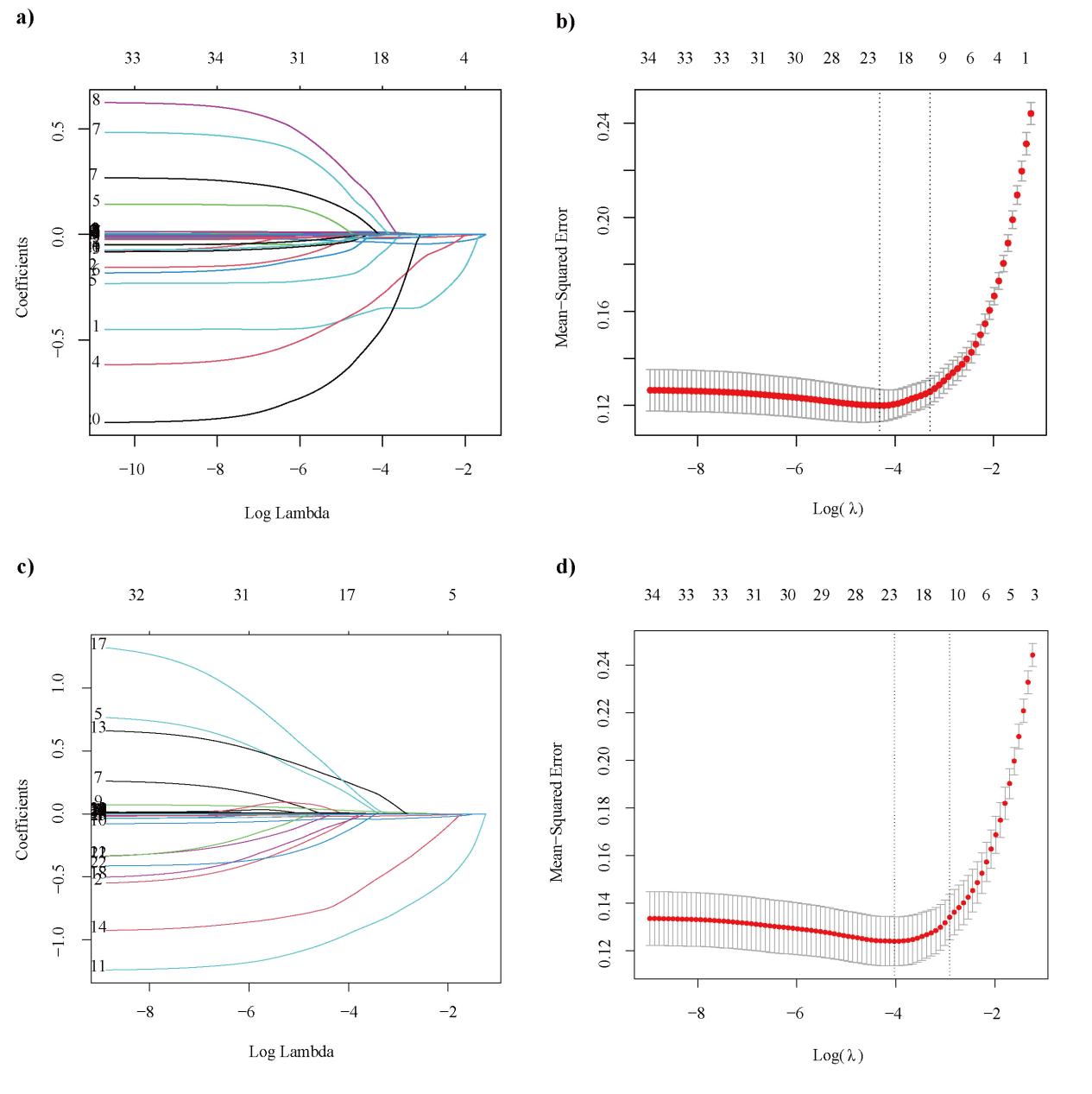


**Supplementary Figure 1.** Application of the LASSO regression model for demographic and clinical feature selection. **a)** and **c)** The coefficient profile was generated based on the logarithmic (lambda) sequence, with non-zero coefficients determined by the optimal lambda value. **b)** and **d)** The optimal parameter (lambda) in the LASSO model was filtered out through 10-fold cross-validation under minimum criteria. The partial likelihood deviation (binomial deviation) curve was plotted against log (lambda). A vertical dashed line at the optimal value was drawn using one SE of minimum criterion (the 1-SE criterion). **a)** and **b)** LASSO analysis for disease (septic shock). **c)** and **d)** LASSO analysis for clinical outcome (30-day mortality). LASSO: least absolute shrinkage and selection operator. SE: standard error.


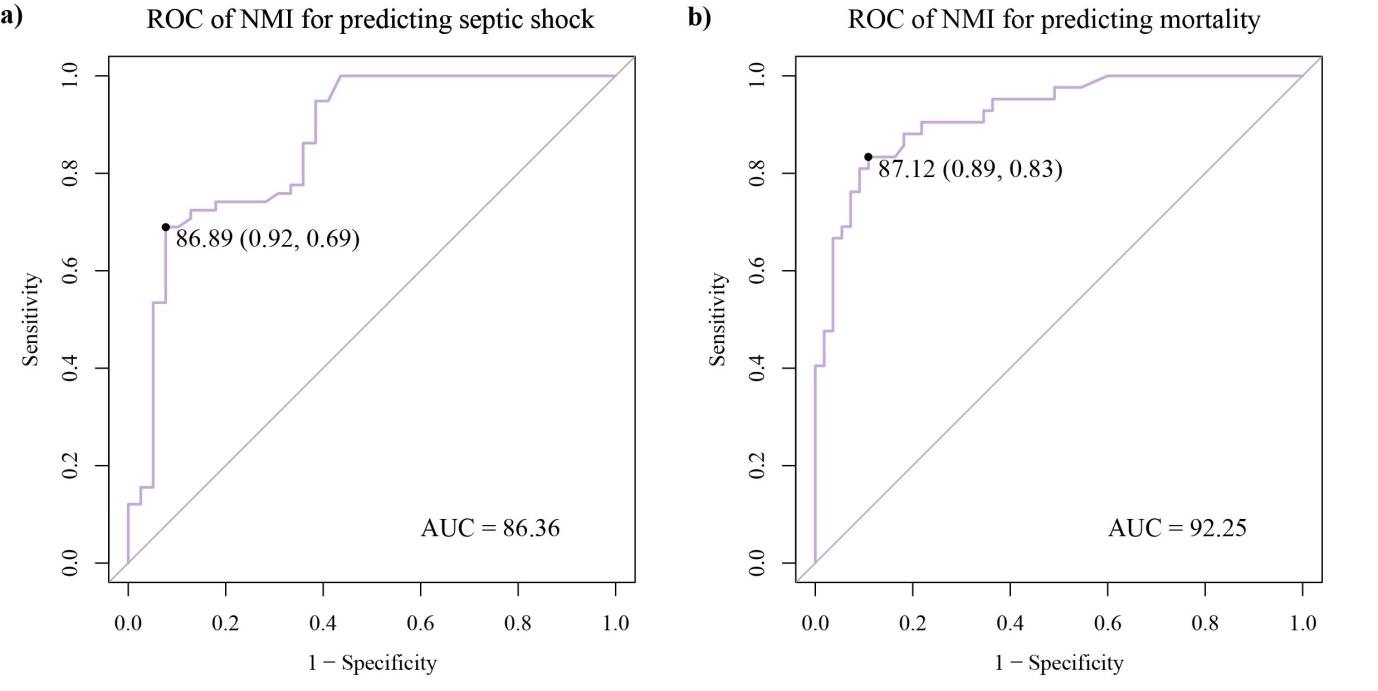


**Supplementary Figure 2.** Diagnostic performance analysis of NMI for predicting the occurrence of septic shock and 30-day mortality. **a)** ROC of NMI for predicting the disease severity. **b)** ROC of NMI for predicting the 30-day mortality. AUC: areas under the ROC curve. NMI: N-myc and STAT interactor. ROC: receiver operating characteristic.


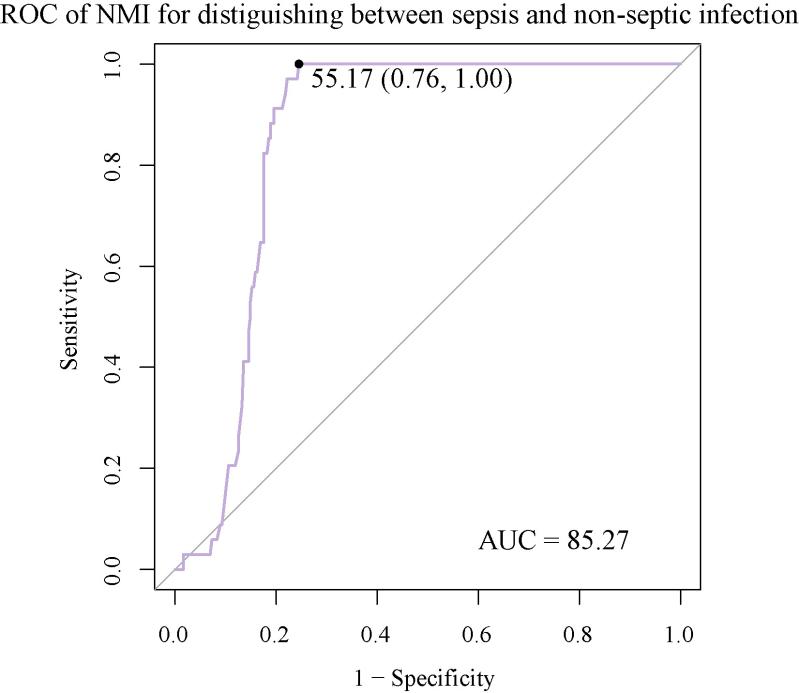


**Supplementary Figure 3.** ROC curve of NMI for distinguishing between sepsis and non-septic infection. AUC: areas under the ROC curve. NMI: N-myc and STAT interactor. ROC: receiver operating characteristic.

## Supplementary Tables

**Table S1.** Comparison of baseline characteristics between patients with sepsis and septic shock

|  |  | Sepsis | Septic shock | *P* value |
| --- | --- | --- | --- | --- |
| n | | 126 | 176 |  |
| Age (y) | | 67 [57, 75] | 66 [58, 76] | 0.877 |
| Sex | |  |  | 0.449 |
|  | Male | 82 (65.1) | 123 (69.9) |  |
|  | Female | 44 (34.9) | 53 (30.1) |  |
| LOS (d) | | 13 [9, 23] | 13 [7, 24] | 0.722 |
| BMI (kg/m^2^) | | 22.3 [19.8, 24.5] | 21.7 [18.3, 24.9] | **0.033** |
| Complication | | 114 (90.5) | 151 (85.8) | 0.296 |
|  | Diabetes mellitus | 33 (26.2) | 52 (29.5) | 0.610 |
|  | Hypertension | 68 (54.0) | 81 (46.0) | 0.213 |
|  | Pulmonary diseases | 13 (10.3) | 14 (8.0) | 0.613 |
|  | Cardiac diseases | 18 (14.3) | 26 (14.8) | 1.000 |
|  | Hepatic insufficiency | 7 (5.6) | 18 (10.2) | 0.215 |
|  | Renal insufficiency | 8 (6.3) | 20 (11.4) | 0.200 |
|  | Cerebrovascular diseases | 20 (15.9) | 32 (18.2) | 0.712 |
|  | Cancer | 34 (27.0) | 38 (21.6) | 0.343 |
| MAP (mmHg) | | 86.8 ± 14.3 | 80.7 ± 15.2 | **<** **0.001** |
| GCS | | 15 [11, 15] | 11 [6, 15] | **<** **0.001** |
| APACHE II | | 15 [11, 21] | 23 [17, 29] | **<** **0.001** |
| SOFA | | 6 [4, 9] | 10 [8, 13] | **<** **0.001** |
| qSOFA | | 1 [0, 1] | 1 [1, 2] | **<** **0.001** |
| Sepsis-related complications | |  |  |  |
|  | Septic cardiomyopathy | 16 (12.7) | 37 (21.0) | 0.085 |
|  | Septic encephalopathy | 0 (0.0) | 2 (1.1) | 0.630 |
|  | Sepsis-induced MOF | 5 (4.0) | 42 (23.9) | **<** **0.001** |
|  | Sepsis-induced renal insufficiency | 36 (28.6) | 114 (64.8) | **<** **0.001** |
|  | Sepsis-induced hepatic insufficiency | 28 (22.2) | 71 (40.3) | **0.001** |
|  | Sepsis-induced respiratory failure | 42 (33.3) | 105 (59.7) | **<** **0.001** |
|  | Sepsis-induced coagulation dysfunction | 15 (11.9) | 53 (30.1) | **<** **0.001** |
| Admission into ICU | | 79 (62.7) | 168 (95.5) | **<** **0.001** |
| Length of staying in ICU (d) | | 4 [0, 10] | 7 [4, 13] | **0.018** |
| Operation | | 42 (33.3) | 71 (40.3) | 0.308 |
| Life support | | 63 (50.0) | 153 (86.9) | **<** **0.001** |
|  | ECMO | 2 (1.6) | 10 (5.7) | 0.134 |
|  | Mechanical ventilation | 60 (47.6) | 146 (83.0) | **<** **0.001** |
|  | CRRT | 14 (11.1) | 92 (52.3) | **<** **0.001** |
| Sites of primary infection | |  |  |  |
|  | Blood infection | 19 (15.1) | 17 (9.7) | 0.210 |
|  | Pulmonary infection | 31 (24.6) | 62 (35.2) | 0.065 |
|  | Urinary infection | 20 (15.9) | 13 (7.4) | **0.032** |
|  | Intestinal infection | 20 (15.9) | 24 (13.6) | 0.706 |
|  | Abdominal infection | 22 (17.5) | 37 (21.0) | 0.533 |
|  | Biliary infection | 2 (1.6) | 7 (4.0) | 0.389 |
|  | Other | 12 (9.5) | 16 (9.1) | 1.000 |
| Laboratory indicators | |  |  |  |
|  | NMI (pg/mL) | 54.2 [45.4, 64.8] | 166.1 [98.8, 437.5] | **<** **0.001** |
|  | PCT (ng/mL) | 4.2 [1.2, 28.6] | 9.7 [3.4, 40.0] | **0.039** |
|  | CRP (mg/L) | 118.8 [68.7, 211.3] | 132.1 [64.9, 215.5] | 0.662 |
|  | IL-6 (pg/mL) | 302.5 [88.8, 1576.5] | 460.0 [169.8, 2179.1] | 0.141 |
|  | Percentage of neutrophils (%) | 88.0 [82.1, 91.9] | 89.7 [84.2, 93.3] | 0.499 |
|  | WBC (× 10^9^/L) | 10.4 [6.7, 15.4] | 10.9 [6.2, 18.1] | 0.062 |
|  | NLR | 14.1 [8.4, 23.5] | 16.3 [7.9, 28.0] | **0.029** |
|  | Lactate (mmol/L) | 1.6 [1.1, 2.3] | 2.6 [1.6, 5.7] | **<** **0.001** |
| Death | | 10 (7.9) | 117 (66.5) | **<** **0.001** |

Data were shown as n (%), mean ± SD, or median [interquartile range].

APACHE II: acute physiology and chronic health evaluation II. BMI: body mass index. CRP: C-reactive protein. CRRT: continuous renal replacement therapy. ECMO: extracorporeal membrane oxygenation. GCS: Glasgow coma scale. ICU: intensive care unit. IL-6: interleukin-6. LOS: length of stay. MAP: mean arterial pressure. MOF: multiple organ failure. NLR: neutrophil-to-lymphocyte ratio. NMI: N-myc and STAT interactor. PCT: procalcitonin. qSOFA: quick sequential organ failure assessment. SD: standard deviation. SOFA: sequential organ failure assessment. WBC: white blood cell. The bolded text indicated *P* < 0.05.

**Table S2.** Comparison of baseline characteristics between survivors and non-survivors within 30 days

|  |  | Survivors | Non-survivors | *P* value |
| --- | --- | --- | --- | --- |
| n | | 175 | 127 |  |
| Age (y) | | 66 [55, 75] | 67 [59, 75] | 0.625 |
| Sex | |  |  | 0.747 |
|  | Male | 117 (66.9) | 88 (69.3) |  |
|  | Female | 58 (33.1) | 39 (30.7) |  |
| LOS (d) | | 14 [9, 23] | 11 [6, 23] | 0.692 |
| BMI (kg/m^2^) | | 22.3 [20.0, 24.8] | 21.8 [18.7, 24.8] | 0.080 |
| Complication | | 151 (86.3) | 114 (89.8) | 0.464 |
|  | Diabetes mellitus | 45 (25.7) | 40 (31.5) | 0.330 |
|  | Hypertension | 90 (51.4) | 59 (46.5) | 0.461 |
|  | Pulmonary diseases | 14 (8.0) | 13 (10.2) | 0.640 |
|  | Cardiac diseases | 23 (13.1) | 21 (16.5) | 0.509 |
|  | Hepatic insufficiency | 8 (4.6) | 17 (13.4) | **0.011** |
|  | Renal insufficiency | 12 (6.9) | 16 (12.6) | 0.134 |
|  | Cerebrovascular diseases | 26 (14.9) | 26 (20.5) | 0.262 |
|  | Cancer | 43 (24.6) | 29 (22.8) | 0.831 |
| MAP (mmHg) | | 84.7 ± 13.7 | 81.2 ± 16.7 | **0.045** |
| GCS | | 15 [11, 15] | 10 [5, 12] | **<** **0.001** |
| APACHE II | | 15 [11, 21] | 25 [20, 31] | **<** **0.001** |
| SOFA | | 7 [4, 9] | 11 [9, 14] | **<** **0.001** |
| qSOFA | | 1 [0, 1] | 1 [1, 2] | **<** **0.001** |
| Sepsis-related complications | |  |  |  |
|  | Septic cardiomyopathy | 26 (14.9) | 27 (21.3) | 0.197 |
|  | Septic encephalopathy | 1 (0.6) | 1 (0.8) | 1.000 |
|  | Sepsis-induced MOF | 10 (5.7) | 37 (29.1) | **<** **0.001** |
|  | Sepsis-induced renal insufficiency | 63 (36.0) | 87 (68.5) | **<** **0.001** |
|  | Sepsis-induced hepatic insufficiency | 38 (21.7) | 61 (48.0) | **<** **0.001** |
|  | Sepsis-induced respiratory failure | 56 (32.0) | 91 (71.7) | **<** **0.001** |
|  | Sepsis-induced coagulation dysfunction | 20 (11.4) | 48 (37.8) | **<** **0.001** |
| Admission into ICU | | 121 (69.1) | 126 (99.2) | **<** **0.001** |
| Length of staying in ICU (d) | | 4 [0, 10] | 8 [4, 17] | **<** **0.001** |
| Operation | | 72 (41.1) | 41 (32.3) | 0.147 |
| Life support | | 93 (53.1) | 123 (96.9) | **<** **0.001** |
|  | ECMO | 3 (1.7) | 9 (7.1) | **0.039** |
|  | Mechanical ventilation | 89 (50.9) | 117 (92.1) | **<** **0.001** |
|  | CRRT | 25 (14.3) | 81 (63.8) | **<** **0.001** |
| Sites of primary infection | |  |  |  |
|  | Blood infection | 24 (13.7) | 12 (9.4) | 0.342 |
|  | Pulmonary infection | 41 (23.4) | 52 (40.9) | **0.002** |
|  | Urinary infection | 29 (16.6) | 4 (3.1) | **<** **0.001** |
|  | Intestinal infection | 22 (12.6) | 22 (17.3) | 0.322 |
|  | Abdominal infection | 35 (20.0) | 24 (18.9) | 0.927 |
|  | Biliary infection | 7 (4.0) | 2 (1.6) | 0.378 |
|  | Other | 17 (9.7) | 11 (8.7) | 0.912 |
| Laboratory indicators | |  |  |  |
|  | NMI (pg/mL) | 59.3 [48.0, 90.2] | 208.7 [113.5, 809.6] | **<** **0.001** |
|  | PCT (ng/mL) | 6.9 [1.3, 40.0] | 8.7 [2.6, 25.4] | 0.331 |
|  | CRP (mg/L) | 120.5 [68.7, 208.0] | 144.1 [64.2, 217.8] | 0.523 |
|  | IL-6 (pg/mL) | 360.7 [76.9, 1954.8] | 402.1 [177.8, 1722.8] | 0.688 |
|  | Percentage of neutrophils (%) | 88.9 [81.5, 92.4] | 89.7 [85.5, 93.0] | 0.446 |
|  | WBC (× 10^9^/L) | 10.4 [6.4, 16.8] | 11.9 [6.6, 17.8] | 0.254 |
|  | NLR | 14.4 [7.3, 25.0] | 15.5 [9.8, 26.1] | 0.133 |
|  | Lactate (mmol/L) | 1.7 [1.1, 2.8] | 2.7 [1.7, 6.2] | **<** **0.001** |
| Septic shock (%) | | 59 (33.7) | 117 (92.1) | **<** **0.001** |

Data were shown as n (%), mean ± SD, or median [interquartile range].

APACHE II: acute physiology and chronic health evaluation II. BMI: body mass index. CRP: C-reactive protein. CRRT: continuous renal replacement therapy. ECMO: extracorporeal membrane oxygenation. GCS: Glasgow coma scale. ICU: intensive care unit. IL-6: interleukin-6. LOS: length of stay. MAP: mean arterial pressure. MOF: multiple organ failure. NLR: neutrophil-to-lymphocyte ratio. NMI: N-myc and STAT interactor. PCT: procalcitonin. qSOFA: quick sequential organ failure assessment. SD: standard deviation. SOFA: sequential organ failure assessment. WBC: white blood cell. The bolded text indicated *P* < 0.05.

**Table S3.** Comparison of baseline characteristics between healthy controls, patients with non-septic infection, and patients with sepsis in the training group

|  |  | Controls | Patients with non-septic infection | Patients with sepsis | *P* value |
| --- | --- | --- | --- | --- | --- |
| n | | 85 | 34 | 302 |  |
| Age (y) | | 67 [58, 74] | 67 [51, 76] | 66 [57, 75] | 0.865 |
| Sex | |  |  |  | 0.929 |
|  | Male | 57 (67.1) | 22 (64.7) | 205 (67.9) |  |
|  | Female | 28 (32.9) | 12 (35.3) | 97 (32.1) |  |
| NMI (pg/mL) | | 11.7 [11.7, 11.7] | 47.1 [45.6, 50.0] | 86.6 [55.7, 208.7] | **<** **0.001** |

Data were shown as n (%), or median [interquartile range].

NMI: N-myc and STAT interactor. The samples below the lower limit were uniformly quantified as half of the minimum detection threshold (11.7 pg/mL).

**Table S4.** Additional diagnostic values of NMI on scores for predicting the septic shock and 30-day mortality of patients with sepsis

|  | Prediction of septic shock | | | |  | Prediction of 30-day mortality | | | |
| --- | --- | --- | --- | --- | --- | --- | --- | --- | --- |
|  | AUC (95% CI) | Specificity (%) | Sensitivity (%) | Youden index |  | AUC (95% CI) | Specificity (%) | Sensitivity (%) | Youden index |
| APACHE II | 0.75 (0.69-0.80) | 70.6 | 70.5 | 0.41 |  | 0.79 (0.74-0.84) | 66.9 | 81.1 | 0.48 |
| NMI+APACHE II | 0.94*** (0.91-0.97) | 97.6 | 77.8 | 0.75 |  | 0.88** (0.84-0.92) | 85.7 | 78.0 | 0.64 |
| SOFA | 0.77 (0.71-0.82) | 81.0 | 58.5 | 0.40 |  | 0.80 (0.75-0.85) | 76.0 | 66.9 | 0.43 |
| NMI+SOFA | 0.93*** (0.90-0.96) | 92.9 | 84.7 | 0.78 |  | 0.87* (0.83-0.91) | 81.7 | 79.5 | 0.61 |
| qSOFA | 0.67 (0.61-0.73) | 85.7 | 31.2 | 0.17 |  | 0.70 (0.61-0.73) | 39.4 | 85.0 | 0.24 |
| NMI+qSOFA | 0.93*** (0.89-0.96) | 88.1 | 88.1 | 0.76 |  | 0.86*** (0.82-0.91) | 85.7 | 76.4 | 0.62 |

APACHE II: acute physiology and chronic health evaluation II. AUC: area under the curve. CI: confidence interval. NMI: N-myc and STAT interactor. qSOFA: quick sequential organ failure assessment. SOFA: sequential organ failure assessment. *: *P* < 0.05, **: *P* < 0.01, ***: *P* < 0.001 compared with the original score systems.

**Table S5.** Comparison of baseline characteristics between the training group and validation group

|  |  | Validation group | *P* value (v.s. training group) |
| --- | --- | --- | --- |
| n | | 97 |  |
| Age (y) | | 68 [60, 77] | 0.131 |
| Sex | |  |  |
|  | Male | 61 (62.9) | 0.433 |
|  | Female | 36 (37.1) |  |
| LOS (d) | | 13 [8, 23] | 0.428 |
| BMI (kg/m^2^) | | 22.3 [20.1, 24.8] | 0.225 |
| Complication | | 77 (79.4) | 0.060 |
|  | Diabetes mellitus | 29 (29.9) | 0.839 |
|  | Hypertension | 53 (54.6) | 0.428 |
|  | Pulmonary diseases | 6 (6.2) | 0.519 |
|  | Cardiac diseases | 11 (11.3) | 0.526 |
|  | Hepatic insufficiency | 7 (7.2) | 0.904 |
|  | Renal insufficiency | 10 (10.3) | 0.917 |
|  | Cerebrovascular diseases | 19 (19.6) | 0.705 |
|  | Cancer | 15 (15.5) | 0.110 |
| MAP (mmHg) | | 81.3 ± 14.5 | 0.303 |
| GCS | | 12 [7, 15] | 0.787 |
| APACHE II | | 20 [13, 24] | 0.617 |
| SOFA | | 8 [6, 11] | 0.430 |
| qSOFA | | 1 [0, 2] | 0.548 |
| Septic shock | | 58 (59.8) | 0.855 |
| Admission into ICU | | 73 (75.3) | 0.208 |
| Length of staying in ICU (d) | | 5 [1, 12] | 0.824 |
| Operation | | 54 (55.7) | 0.274 |
| Life support | | 62 (63.9) | 0.197 |
|  | ECMO | 6 (6.2) | 0.527 |
|  | Mechanical ventilation | 56 (57.7) | 0.077 |
|  | CRRT | 28 (28.9) | 0.314 |
| Laboratory indicators | |  |  |
|  | NMI (pg/mL) | 86.0 [51.5, 108.7] | 0.088 |
|  | PCT (ng/mL) | 6.4 [2.1, 36.6] | 0.714 |
|  | CRP (mg/L) | 157.1 [76.0, 226.4] | 0.372 |
|  | IL-6 (pg/mL) | 707.0 [160.0, 3561.5] | 0.289 |
|  | Percentage of neutrophils (%) | 90.3 [83.7, 93.4] | 0.833 |
|  | WBC (× 10^9^/L) | 11.5 [7.3, 15.2] | 0.723 |
|  | NLR | 18.2 [8.3, 28.6] | 0.519 |
|  | Lactate (mmol/L) | 1.9 [1.4, 3.2] | 0.100 |
| Death | | 42 (43.3) | 0.922 |

Data were shown as n (%), mean ± SD, or median [interquartile range].

APACHE II: acute physiology and chronic health evaluation II. BMI: body mass index. CRP: C-reactive protein. CRRT: continuous renal replacement therapy. ECMO: extracorporeal membrane oxygenation. GCS: Glasgow coma scale. ICU: intensive care unit. IL-6: interleukin-6. LOS: length of stay. MAP: mean arterial pressure. NLR: neutrophil-to-lymphocyte ratio. NMI: N-myc and STAT interactor. PCT: procalcitonin. qSOFA: quick sequential organ failure assessment. SD: standard deviation. SOFA: sequential organ failure assessment. WBC: white blood cell. The detailed data of the training group was shown in Table 1 in the article.
